# Association between bisphenol A exposure and body mass index in Chinese school children: a cross-sectional study

**DOI:** 10.1186/1476-069X-11-79

**Published:** 2012-10-19

**Authors:** He-xing Wang, Ying Zhou, Chuan-xi Tang, Jin-gui Wu, Yue Chen, Qing-wu Jiang

**Affiliations:** 1 Key Laboratory of Public Health Safety of Ministry of Education, School of Public Health, Fudan University, Shanghai, 200032, China; 2 Centers for Disease Control and Prevention of Changning district, Shanghai, 200051, China; 3 Department of Epidemiology and Community Medicine, Faculty of Medicine, University of Ottawa, Ottawa, Ontario, Canada

**Keywords:** Bisphenol A, Urine, Body mass index, Obesity, School children

## Abstract

**Background:**

There is increasing evidence suggesting that Bisphenol A (BPA), one of the highest volume chemicals produced worldwide, can interfere with the body’s natural weight control mechanisms to promote obesity. However, epidemiological studies for this are limited, especially for children.

**Methods:**

A cross-sectional study was conducted to investigate the association between BPA exposure and body mass index (BMI) in school children. Three primary and three middle schools were randomly selected from 26 primary and 30 middle candidate schools in Changning District of Shanghai City in China. According to the BMI-based criteria by age and sex for screening of overweight or obese children, we randomly chose 20 obese, 10 overweight, and 30 normal weight children aged 8-15 years of age from each selected school. First morning urine was collected and total urine BPA concentrations were determined by ultra-performance liquid chromatography tandem mass spectrometry. Multiple linear regression analysis was conducted to examine the association of urine BPA concentrations and daily intake estimates with BMI.

**Results:**

BPA was detected in 84.9% of urine samples with a geometric mean of 0.45 ng/mL. The daily intake estimates ranged from 0.03 μg/day to 1.96 μg/day with a geometric mean of 0.37 μg/day. The average urine BPA concentrations and daily intake estimates were similar for boys and girls, but significantly higher in older children than younger ones, and showed an increasing trend with BMI. Multiple linear regression analyses showed that urine BPA concentrations were significantly associated with increasing BMI values in all subjects after adjustment for age and sex and the results were similar before and after corrected by urine specific gravity. When stratified by age or sex, the associations remained significant in females and in those 8-11 years of age before corrected by specific gravity. Similar results were shown for the association between BMI and daily intake estimates.

**Conclusions:**

There is a possibility that BPA exposure increases BMI in school children. Given the cross-sectional nature of this study, longitudinal studies are warranted to confirm BPA exposure as a contributor to increased BMI in children.

## Background

Bisohenol A (BPA) is one of the highest volume chemicals produced worldwide, with over six billion pounds produced each year [1]. It was mainly used for the production of epoxy resins and polycarbonate plastics. Epoxy resins are commonly used as food-contact surface lacquer coatings and polycarbonate plastics are used in compact disk manufacturing, household appliances, food packaging and plastic bottles among others [2]. Because unbound monomers remain after BPA polymerization, BPA molecules can be leached from those products into environment and food. The widespread occurrence of BPA in environment and food has been well documented [3]. The main route of human exposure to BPA is through food intake and BPA has been detected in human urine, blood, milk and tissues, indicating that human exposure to BPA is extensive [4]. Moreover, lab researches have found that BPA is a typical endocrine disrupting chemical (EDC) [5], and human exposure to BPA has been reported to be associated with various adverse effects, such as metabolic and reproductive diseases [6].

There is increasing evidence suggesting that some endocrine disrupting chemicals can act as obesogens and interfere with body’s natural weight control mechanisms by disrupting adipose tissue biology, endocrine hormone systems, or central hypothalamic-pituitary-adrenal axis [7,8]. However, most of the studies about the association of BPA, one of focused EDCs, with obesity are lab researches [9,10]. For example, Huc et al observed that lipid accumulation in HepG2 cells was induced by BPA at 10^-4^-10^-12^ M level [11]. Sargis et al. reported that BPA significantly stimulated glucocorticoid receptor (GR) to promote adipogenesis without significant activation of the peroxisome proliferator-activated receptor-γ in the 3 T3-L1 Cell Line at an incubation concentration of 100 nM [12]. Gestational or perinatal exposure of mice to 2.4-500 μg/kg body weight -day of BPA resulted in increased body weight of offspring [13-15].

To our knowledge, there have been only three published epidemiological studies primarily designed for the association between BPA exposure and obesity [16-18]. Two of them were conducted in adults, one in children and adolescents, and all of them used a cross-sectional design. Children and adolescents are in rapid growth and are more susceptible to external disturbance compared with adults. Additionally, it has been reported that children are extensively exposed to BPA and exposure levels are even higher compared with adults [19,20]. In this study we examined the association between BPA exposure and body mass index (BMI) in Chinese school children.

## Methods

### Study population

There were a total of 26 primary schools with 17,570 students and 30 middle schools with 21,059 students in Changning District in Shanghai City of China. For the current study, three primary schools (a total of 518, 448, and 573 students, respectively) and three middle schools (467, 541, and 374 students, respectively) were randomly selected. From each selected school, 20 obese, 10 overweight, and 30 normal weight students aged 8-15 years were randomly chosen in the basis of the most recent yearly regular physical examination conducted in October, 2011, and available information included weight, height, age, sex, and ethnicity of students. The students with liver, kidney, or endocrine diseases were excluded. A total of 360 students were selected and they were all Han people. The study was approved by the Ethical Review Board of Fudan University School of Public Health. Either participants or guardians provided a written informed consent for study participation.

### Anthropometric measurement

Body weight (kg) and height (cm) were measured using the same type of apparatus and followed the standard procedures recommended by Cameron [21]. Subjects were required to wear light clothes and stand straight and barefoot when being measured. All instruments were properly calibrated. Each technician was required to pass a training course for the anthropometric measurement. BMI is a reasonable measure of fatness in children and adolescents [22] and calculated as weight in kilograms divided by height in meters squared. Normal weight, overweight, and obese individuals were identified according to the BMI-based criteria by age and sex proposed by the Working Group on Obesity in China (WGOC) (see Additional file 1: Table S1 for the BMI cut-off values) [23].

### Measurement of urine BPA

First morning urine was collected by participants themselves. In order to minimize the contamination of BPA during urine sampling and storing, the participants were told to avoid a contact of urine with plastic products in the process of urine collection, and urine samples were collected in glass centrifuge tubes, which was rinsed by acetone and baked at 350°C for 2 hours. The collected urine samples were transported to lab as soon as possible and stored in dark at -20°C until analysis. All the urine samples were collected between January 3 and 6, 2012, about 3 months after the latest physical examination.

The analysis of total urine BPA (free and conjugated) was carried out by solid-phase extraction coupled with ultra-performance liquid chromatography isotope dilution tandem mass spectrometry, as described previously [24,25]. All urine analyses were carried out in a random order for three BMI groups (normal weight, overweight, and obesity), which was blinded to analytical technicians. To reduce a potential systematic drift of analytical method over time, all the analyses of urine samples were completed in March 2012. The analytical method was included in the Supplement.

### Statistical analyses

For measurements below the limit of detection (LOD, 0.07 ng/mL, see Additional file 1), a default value of LOD divided by the square root of 2 was assigned, which produces reasonably nonbiased means and standard deviations [26]. Daily intake estimates were calculated in the basis of urine BPA concentrations using a modified method proposed by Lakind and Naiman [27], with the reference values recommended by the International Commission on Radiological Protection (ICRP) [28]. The average value (600 ml/day) of the ICRP urine outputs (500 ml/day for children 5 years of age and 700 ml/day for those 10 years of age) was used for the 6-11 year age group. For the 12-19 year age group, the ICRP urine output of 15 years of age (1200 ml/day) was used. The calculation formula is DI = C × V/1000, where DI is the daily intake estimate (μg/day), C is the urinary BPA concentration (ng/mL), and V is urinary output (mL/day).

For descriptive statistics, arithmetic mean and standard deviation for weight, height, and BMI for all subjects or by sex, age (8-9, 10-11, 12-13, and 14-15 years of age), and BMI (normal weight, overweight, and obesity) were calculated. For urinary BPA concentrations and daily intake estimates, we also calculated geometric mean (GM) and its 95% confidence interval (CI), median, and range. The 95% CI of GM was a natural exponential transform of that of arithmetic mean of naturally log-transformed urine BPA concentrations. A natural log-transformation was used to normalize the BPA data. Analysis of variance was used to examine the associations of BPA with age, sex, and BMI.

Due to the glucuronidation of BPA in the liver and its elimination by active tubular secretion, creatinine adjustment may not be appropriate for urine BPA concentration [29,30]. Additionally, BMI could predict the urine creatinine concentration, and therefore, the associations between urine BPA and BMI may be altered by creatinine adjustment [31]. Instead, we used specific gravity (SG) to correct for urinary dilution as recommended by Mahalingaiah et al. [30]. SG was measured using a handheld refractometer (Atago PAL 10-S; Tokyo, Japan). The correction formula was P_c_ = P × [(1.024–1)/(SG–1)], where P_c_ is the SG-corrected BPA concentration (ng/mL), P is the experimental BPA concentration (ng/mL), and SG is the specific gravity of urine sample [32].

Since the distribution of BMI values was slightly right skewed, a natural log-transformation was used to improve the normal distribution of BMI values. And then multiple linear regression analyses were performed to study the associations of naturally log-transformed urinary BPA concentrations or daily intake estimates with BMI values. We first included all the subjects in regression models and then conducted age- and sex-stratified analysis before and after taking into account for covariates. Urine BPA concentrations and daily intake estimates were examined before (adjusted model 1) and after (adjusted model 2) SG correction.

Analyses of variance for mean differences in BMI and their 95% CIs between quartile 2, 3 or 4 and quartile 1 (reference) of urine BPA concentration were carried out in all subjects or stratified by age and sex taking either age in year or sex into consideration. Data analyses were performed using SPSS (version 17; SPSS, Inc., Chicago, IL, USA); p < 0.05 was considered significant, and all statistical tests were two-sided.

## Results

Of 360 eligible subjects, 53 did not provide urine samples and 48 provided urine samples but not first morning urine. A total of 259 subjects were included in this study, and among them, 124 had normal weight, 53 were overweight, and 82 were obese (Table 1). The mean and standard deviation of weight, height, and BMI in all subjects or by age or sex were listed in Table 1.

**Table 1:** Mean and standard deviation of weight, height, and body mass index (BMI) among study participants

**Group**	**No. (%)**	**Weight (kg)**	**Height (m)**	**BMI (kg/m**^**2**^**)**
All	259 (100.0)	47.4 ± 16.5	1.47 ± 0.13	21.3 ± 4.6
Age (years)				
8-9	64 (24.7)	32.5 ± 9.3	1.31 ± 0.06	18.7 ± 4.0
10-11	80 (30.9)	43.6 ± 11.2	1.45 ± 0.08	20.5 ± 3.7
12-13	75 (29.0)	54.6 ± 14.9	1.55 ± 0.09	22.3 ± 4.4
14-15	40 (15.4)	65.6 ± 12.9	1.62 ± 0.07	25.0 ± 4.6
Sex				
Female	129 (49.8)	47.3 ± 15.1	1.48 ± 0.12	21.1 ± 4.3
Male	130 (50.2)	47.6 ± 17.9	1.46 ± 0.14	21.4 ± 4.9
BMI				
Normal weight	124 (47.9)	36.7 ± 10.9	1.44 ± 0.14	17.4 ± 2.1
Overweight	53 (20.4)	52.8 ± 12.5	1.50 ± 0.13	23.0 ± 1.9
Obesity	82 (31.7)	60.1 ± 15.3	1.50 ± 0.12	26.0 ± 3.2

As shown in Table 2, BPA was detected in 84.9% of urine samples with a GM of 0.45 ng/mL and a median of 0.60 ng/mL. The daily intake estimates ranged from 0.03 μg/day to 1.96 μg/day with a GM of 0.37 μg/day. The average levels of the urine BPA concentrations and the daily intake estimates were similar in boys and girls but were significantly higher in older children than younger ones. Urine BPA concentration and daily intake estimates increased with BMI after adjustment for sex and age. A pair comparison showed that average urine BPA concentrations and daily intake estimates were significantly higher in the obesity group compared with the normal weight group. The adjusted mean difference was 0.658 (95% CI: 0.195, 1.120, p = 0.006) for naturally log-transformed urine BPA and was 0.642 (95% CI: 0.161, 1.122, p = 0.009) for naturally log-transformed daily intake estimate. However, there were no significant difference between the overweight group and the normal weight group or between the obesity group and the overweight group.

**Table 2:** Urine bisphenol A (BPA) concentrations (ng/mL) and daily intake estimates (μg/day) among study participants

	**Group**	** Geometric mean (95%CI)**		** Percentile (not corrected by SG)**					**p-Value**
		Corrected by SG	Not corrected by SG	Minimum	25%	50%	75%	Maximum	
Urine BPA	All	0.40 (0.33, 0.49)	0.45 (0.37, 0.55)	0.05	0.2	0.6	1.37	16.3	
Daily intake estimate	All	0.33 (0.27, 0.41)	0.37 (0.29,0.45)	0.03	0.12	0.48	1.26	1.96	
	Age (years)								
Urine BPA	8-11	0.32 (0.25, 0.43)	0.31 (0.23, 0.41)	0.05	0.1	0.35	1.05	14.00	P<0.001^a^
	12-15	0.53 (0.40, 0.70)	0.72 (0.54, 0.94)	0.05	0.35	0.85	1.91	16.3	
Daily intake estimate	8-11	0.19 (0.15, 0.25)	0.19 (0.14,0.24)	0.03	0.06	0.21	0.63	8.64	P<0.001^a^
	12-15	0.63 (0.48, 0.84)	0.86 (0.65,1.13)	0.06	0.42	1.02	2.30	19.56	
	Sex								
Urine BPA	Female	0.43 (0.33, 0.58)	0.46 (0.35, 0.63)	0.05	0.2	0.55	1.48	16.30	P = 0.812^b^
	Male	0.38 (0.29, 0.50)	0.43 (0.33, 0.58)	0.05	0.2	0.63	1.2	10.90	
Daily intake estimate	Female	0.35 (0.26, 0.48)	0.38 (0.28,0.52)	0.03	0.12	0.48	1.47	19.56	P = 0.939^b^
	Male	0.31 (0.23, 0.41)	0.35 (0.26,0.48)	0.03	0.15	0.48	1.20	13.08	
	BMI								
Urine BPA	Normal weight	0.33 (0.25, 0.45)	0.33 (0.25, 0.45)	0.05	0.1	0.35	1.05	14.40	P = 0.018^c^
	Overweight	0.37 (0.24, 0.58)	0.47 (0.30, 0.74)	0.05	0.16	0.78	1.35	11.00	
	Obesity	0.57 (0.42, 0.78)	0.68 (0.49, 0.95)	0.05	0.35	0.78	1.79	16.30	
Daily intake estimate	Normal weight	0.26 (0.19, 0.36)	0.26 (0.19,0.36)	0.03	0.06	0.30	1.08	10.26	P = 0.032^c^
	Overweight	0.33 (0.20, 0.53)	0.41 (0.25,0.69)	0.03	0.13	0.56	1.49	13.20	
	Obesity	0.47 (0.34, 0.65)	0.56 (0.40,0.80)	0.03	0.29	0.66	1.59	19.56	

Abbreviation: CI (confidence interval), SG (specific gravity).

^a^ Analysis of variance for urine BPA and daily intake estimate uncorrected by SG adjusted for sex.

^b^ Analysis of variance for urine BPA and daily intake estimate uncorrected by SG adjusted for age.

^c^ Analysis of variance for urine BPA and daily intake estimate uncorrected by SG adjusted for age and sex.

Table 3 showed that urine BPA concentration was significantly associated with increasing BMI as a continuous variable in all subjects after adjustment for age and sex. The results were similar before and after urine BPA concentrations were corrected by SG. There were sex- and age- related variations in the association. Adjusted model 1 shows that before corrected by SG, the association between urine BPA concentration and BMI was significant in females and in the 8-11 year age group. However, the association was not significant after corrected by SG although the association in the 8-11 age group narrowly missed statistical significance (p = 0.081) (adjusted model 2). The regression coefficient (β) represented the change of naturally log-transformed BMI value per one unit of naturally log-transformed urine BPA concentration. The results of BPA intake estimate associated with BMI were similar to those of urine BPA associated with BMI (see Additional file 1: Table S2).

**Table 3:** **Multiple linear regression analyses for the associations between urine bisphenol A (BPA) concentration (ng/mL) and body mass index (BMI, Kg/m**^**2**^**) among study participants**

**Group**	**Crude analysis**^**a**^	**Adjusted model 1**^**b**^	**Adjusted model 2**^**c**^
	**β ^d^ (95% CI)**	**β (95% CI)**	**β (95% CI)**
All	0.019 (0.002, 0.035)	0.018 (0.004, 0.032)	0.017 (0.002, 0.032)
	P = 0.027	P = 0.011	P = 0.026
Age (years)			
8-11	0.010 (-0.010, 0.030)	0.020 (0.001, 0.038)	0.017 (-0.002, 0.037)
	P = 0.330	P = 0.041	P = 0.081
12-15	0.013 (-0.012, 0.039)	0.020 (-0.003, 0.044)	0.019 (-0.005, 0.044)
	P = 0.306	P = 0.088	P = 0.118
Sex			
Female	0.021 (-0.001, 0.043)	0.020 (0.002, 0.038)	0.015 (-0.004, 0.034)
	P = 0.065	P = 0.028	P = 0.115
Male	0.017 (-0.008, 0.042)	0.016 (-0.007, 0.038)	0.018 (-0.005, 0.041)
	P = 0.188	P = 0.164	P = 0.123

Abbreviation: CI (confidence interval).

^a^Included a variable of urine BPA corrected for specific gravity.

^b^Not corrected urine BPA concentrations by specific gravity and included the covariables of age and sex.

^c^Corrected urine BPA concentrations by specific gravity and included the covariables of age and sex.

^d^Regression coefficient.

**Figure 1:**
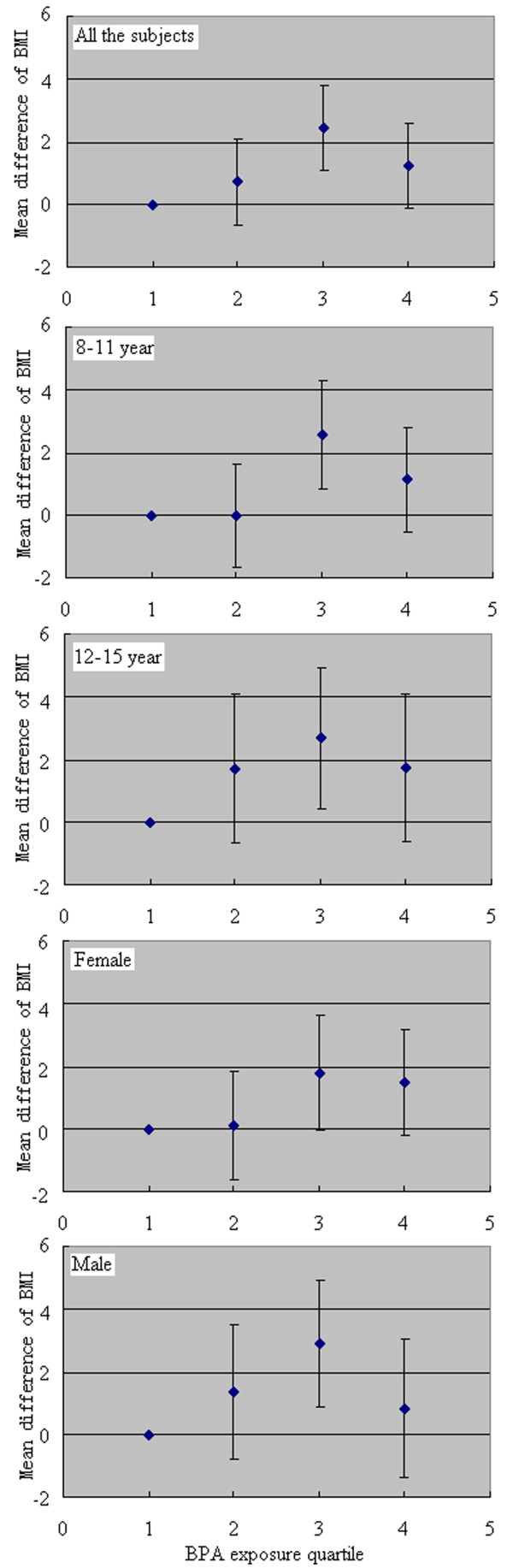
**Analysis of variance for mean differences of body mass index (BMI) between quartiles 2, 3 or 4 of urine BPA concentrations corrected by specific gravity with quartile 1 in all subjects or stratified by age (8-11 and 12-15 year age groups) or sex after adjustment for age in year and sex.** Error bars indicated 95% confidence interval.

Figure 1 shows the adjusted mean differences in BMI for urine BPA concentration quartiles 2, 3, and 4 compared with quartile 1. Average BMI tended to be higher in the BPA quartile 2, 3 and 4 groups compared with the quartile 1 group.

## Discussion

Only few epidemiological studies have been conducted to determine the association between BPA exposure and obesity, and provided inconsistent results. Two cross-sectional studies found no significant associations: one used data from the InCHINTI Study, a prospective population-based study of Italian adults 20-74 years of age [33], and another used data from the National Health and Nutrition Examination Survey (NHANES) 2003-2004 of adults 18-74 years of age [34]. Other three cross-sectional studies found a significant positive association between BPA exposure and obesity based on combined data from the NHANES 2003-2004 and the NHANES 2005-2006 [16], data from children and adolescents who participated in the NHANES 2003-2008 [17], and data from Chinese adults 40 years of age or older [18]. The discrepancy between the studies conducted by Lang et al. [34] and Carwile et al. [16] might reflect a different statistical power to detect a moderate association. In contrast to the studies described above, our study’s participants were school children, and the analysis of BPA was performed in a short time span (one month), in the same laboratory, and by the same analytical team, which minimized potential measurement biases.

The levels (0.45 ng/m L of GM) and detection frequency (84.9%) of BPA in this study were below those from most previous studies of children and adolescents (Table 4). The detection frequency of BPA in these studies ranged from 92.9% to 98.6% with an exception of 79% in a study of Egypt girls [35], and the levels varied between 0.84 ng/m L and 3.7 ng/m L of GM. Based on individual body weights, we calculated BPA daily intake estimates, which ranged from 0.19 ng/kg-day to 277.30 ng/kg-day with a GM of 8.22 ng/kg-day (detail data available upon request). The GM of daily intake estimates in this study was also lower than those from most previous studies (Table 4), and much lower than the tolerable daily intake of 50 μg/kg-day recommended by the European Food Safety Authority (EFSA) in 2007 [36]. The GM of daily intake estimates in the previous studies ranged from 54.0 ng/kg-day to 71.0 ng/kg-day (Table 4).

**Table 4:** Urine bisphenol A (BPA) concentrations (ng/mL) and daily intake estimates (ng/kg-day) from previous studies

**Ref**	**Country**	**Sample size (male/female)**	**Age (years)**	**Sampling date (year)**	**Detection frequency**	**BPA concentration**	**Daily intake estimate**
[19]	Canada	1031(524/507)	6-11	2007-2009	93.20%	GM 1.30	-
		945(489/456)	12-19		93.80%	GM 1.50	-
[24]	China	17(10/7)	≤19	2010	94.3% for all	GM 1.65	Median 2.13 μg/day for all
[27]	U.S.	314	6-11	2003-2004	92.6% for all	GM 3.5	GM 64.6
		715	12-19			GM 3.7	GM 71.0
[35]	Egypt	57 girls	10-13	2009	79%	GM 0.84	-
[36]	U.S.	356	6-11	2005-2006	92.9% for all	GM 2.9	GM 54.0
		702	12-19			GM 2.5	GM 48.0
[37]	German	599	3-14	2003-2006	98.60%	GM 2.66	GM 60
[38]	Spanish	30 boys	4	2005-2006	96.70%	Median 4.2	-

Abbreviation: (GM) Geometric mean, (U.S.) United States.

-: Not reported.

In the present study, multiple linear regression analysis showed that high urine BPA and daily intake estimate were significantly associated with an increase in BMI value in all subjects. Specific gravity correction weakened the association, especially after stratified by age or sex. It was reported that overweight and obese subjects might have less urine output [39], which might lead to a higher urine BPA concentration, and similarly a higher urine SG. In this respect, urine SG correction was appropriate. However, there is also a possibility that urine solute excretion increases urine SG in overweight or obese subjects [40], and urine SG correction might result in an underestimation of true urine BPA concentrations in overweight or obese subjects and therefore weaken the association with BMI. Small sample size could be another important reason for non-significant association when stratified by sex and age.

Animal and cell studies have demonstrated that BPA can promote adipogenesis by diverse mechanisms [7,8]. Among them, BPA interactions with specialized nuclear receptors (such as steroid hormone receptors or thyroid hormone receptors) may serve as a primary mechanism for promoting adipogenesis [41].

BPA has been reported to result in a variety of dysfunctions by binding to estrogen receptors (ERs) [6]. Soriano et al. found that BPA behaved as a strong estrogen via nuclear ERβ by using pancreatic β-cell from ERβ-/- mice at 1 nM of BPA exposure level [42]. BPA also stimulated calcium influx and prolactin secretion in rat pituitary tumor cells by membrane estrogen receptor α at as low as pM level [43]. Three human cell lines (Ishikawa, HeLa and HepG2) were used to evaluate the estrogenic promoter activity of BPA on estrogen receptors α and β, and BPA were found to act as cell type specific agonists or antagonists at different concentration levels ranging from 1 nM to 1000 nM [44]. Moreover, a role for sex steroid hormones in regulating adipocyte hypertrophy and hyperplasia was confirmed by knockout models of sex steroid pathway components [7].

Thyroid function is critical to the maintenance of basal metabolism, and BPA may influences thyroid hormone. A large cross-sectional study from Denmark found that thyroid function,was associated with BMI [45]. Fox et al. found that thyroid function within the reference range, as assessed by serum thyroid stimulating hormone concentration, is associated with body weight in both sexes using data from Framingham Offspring Study [46]. Moreover, Moriyama et al. observed that BPA could inhibit the binding of T3 to the rat hepatic thyroid hormone receptors with an inhibition constant (Ki) of 200 μM and suppress the transcriptional activities mediated by endogenous thyroid hormone receptors at as low as 100 nM [47].

In our studies, we observed that there are some age- or sex-specific differences in the associations between urine BPA and BMI. Previous epidemiological studies also reported age- or sex-specific differences in the association between urine BPA and obesity or between urine phthalate metabolites and BMI [16,48]. Different sex hormone profiles between sex and age subgroups might explain the differences observed. Puberty usually begins between 10 and 13 years of age, and serum levels of endogenous sex hormones in puberty significantly rise, especially estradiol in girls and testosterone in boys. There also exist distinct differences in sex hormones between prepubertal boys and girls. Klein et al. reported that prepubertal girls had a 8-fold higher estrogen level than prepubertal boys [49]. The differences may lead to different susceptibility of participants to sex hormone perturbation caused by BPA.

Although plausible physiological mechanisms for BPA as an obesogen are present, there are some important gaps between our study and lab researches for meaningful interpretations. In this study, the GM of daily intake estimates was 8.22 ng/kg-day, but it was approximately 290-60,000 times lower than the pre or perinatal exposure dose (2.4-500 μg/kg-day) tested in mice lab studies reported previously [13-15]. Moreover, based on a human test about BPA metabolism and kinetics [29], the average concentration level of free serum BPA in study participants was estimated at pM level using the average BPA exposure level (8.22 ng/kg-day) in this study. This estimated serum BPA level was comparable to the least effect concentration inducing lipid accumulation in the HepG2 cell [11], but about 50,000 times lower than that promoting adipogenesis in 3 T3-L1 cell [12]. The reasons for the discrepancy of detected effect level are not clear, and the impacting factors may include cross-species, different exposure window during life course, different exposure route and/or duration to BPA for animal studies and different cell types or effect endpoints tested for cell experiments.

There were some study limitations that should be considered when interpreting the results. First, the cross-sectional nature of the association of BPA and obesity could not rule out the possibility of reverse causation. BPA exposure could be a proxy for other variables related to obesity, such as caloric intake, diet composition, daily indoor time, parental education, and family income level [17]. Of them, caloric intake or diet composition is of concern. Because food is thought as the main exposure route for BPA in human population, it is possible that increased overall calaric intake or different diet composition among obese individuals might increase the risk of BPA exposure, compared to normal weight ones.

Second, due to the short half-life (less than 6 h) of BPA in human body [29], the one spot urine analysis is not an ideal measure for long-term exposure given that obesity was most likely associated with long-term low-dose exposure. Mahalingaiah et al. reported that spot urine samples showed a moderate sensitivity for predicting an individual’s tertile categorization. Misclassification due to this spot urine might result in an attenuated estimate of the strength of association between BPA exposure and BMI [30]. The first morning urine used in the current study improved this circumstance by adding more comparability between individuals than pure spot urine as most previous studies did. However, due to its rapid metabolism in human body, BPA exposure estimates from first morning urine may just represent the exposure at the prior meal (dinner), not daily or average exposure level. Given the food indigestion as the main exposure route to BPA, the use of first morning urine may result in an elevated BPA exposure estimate [50,51].

Third, exposure to BPA might also be an indicator of exposure to multiple environmental chemicals such as phthalates and organotins. Some studies have reported that urine phthalate metabolites were significantly associated with BMI [52]. Urine concentrations of other environmental chemicals, especially phthalate metabolites and organotins, may be needed to be investigated to clarify their roles in the association between BPA exposure and BMI.

## Conclusions

In this study, we found that urine BPA concentration and daily BPA intake estimate were positively associated with BMI in Chinese school children. Due to being much lower than the recommended tolerable daily intake of 50 μg/kg-day proposed by EFSA, the effective dose estimated from the current cross-sectional data required a further risk reassessment of BPA exposure. Given the cross-sectional nature of this study, longitudinal studies are warranted to confirm BPA exposure as a contributor to increased BMI.

## Abbreviations

BPA: Bisphenol A; BMI: Body mass index; EDCs: Endocrine disrupting chemicals; GR: Glucocorticoid receptor; WGOC: Working group on obesity in China; LOD: Limit of detection; ICRP: International commission on radiological protection; CI: Confidence interval; GM: Geometric mean; SG: Specific gravity; EFSA: European food safety authority; ERs: Estrogen receptors; NHANES: National health and nutrition examination survey; ER: Estrogen receptor.

## Competing interests

All the authors declared that they had no competing interests.

## Authors’ contributions

HXW and YZ had full access to all of the data in the study and take responsibility for the integrity of the data and the accuracy of the data analysis; YZ, YC, and QWJ developed the study concept and design; CXT, JGW, HXW, and YZ performed the collection of anthropometric data and urine samples; HXW, YZ, YC, and QWJ conducted the analysis and interpretation of data; CXT, JGW, HXW, YZ, and YC wrote the majority of the manuscript; YC and QWJ had critical revision of the manuscript for important intellectual content; HXW, YZ, and YC conducted the statistical analysis; YZ, JGW, and QWJ supplied administrative, technical, or material support; YC and QWJ supervised the study. All authors read and approved the final manuscript.

## Supplementary Material

Additional file 1 **Table S1.** BMI-based criteria by age and sex for screening of overweight and obese school children proposed by Working Group on Obesity in China (WGOC) in 2005. **Table S2** Multiple linear regression analyses for the associations between daily intake estimates (μg/day) and body mass index (BMI, Kg/m^2^) among study participants. Click here for file
