# Supplementary material for: Association between bisphenol A exposure and body mass index in Chinese school children: a cross-sectional study
Source: Environ Health. 2012 Oct 19;11:79. doi: 10.1186/1476-069X-11-79 (PMC3549797; doi:10.1186/1476-069X-11-79)
Supplement: Additional file 1 — Table S1. BMI-based criteria by age and sex for screening of overweight and obese school children proposed by Working Group on Obesity in China (WGOC) in 2005. Table S2 Multiple linear regression analyses for the associations between daily intake estimates (μg/day) and body mass index (BMI, Kg/m2) among study participants. [file 1476-069X-11-79-S1.doc]

**Supporting Information**

**Association between bisphenol A exposure and body mass index in Chinese school children: A cross-sectional study**

He-xing Wang,^1^ Ying Zhou, ^1*^ Chuan-xi Tang, ^2^ Jin-gui Wu,^2^ Yue Chen, ^3^ Qing-wu Jiang^1^

^1^ Key Laboratory of Public Health Safety of Ministry of Education, School of Public Health, Fudan University，Shanghai, 200032, China

^2^ Centers for Disease Control and Prevention of Changning district, Shanghai, 200051, China

^3^ Department of Epidemiology and Community Medicine, Faculty of Medicine, University of Ottawa, Ottawa, Ontario, Canada

**Chemicals and Reagents**

Bisphenol A (BPA) (＞99 in purity), bisphenol A-d_16_ (＞95 in purity), β-glucuronidase/sulfatase from *Helix pomatia* (Type H-2，[≥100,000 units glucuronidas /mL and ≤7,500 units sulfatase /mL](http://www.sigmaaldrich.com/catalog/product/sigma/g7017?lang=zh&region=CN) ), 4-methylumbelliferone glucuronide (＞97 in purity), acetic acid, and ammonium acetate were purchased from Sigma-Aldrich (Shanghai, China). 4-thylumbelliferone and 4-methylumbelliferone-13C_4_ were obtained from Cambridge Isotope Laboratories (MA, USA). Reagents of LC-MS grade, including water, methanol and acetonitrile, were from Fisher (Fair Lawn, NJ, USA). Deionized water was from Milli-Q-Plus (Millipore, Bedford, MA, USA) and only used for sample pretreatment.

**Sample Preparation**

An aliquot (1.0 mL) of urine was spiked with 50 μL of standard solution containing 200 ng/mL of BPA-d_16_, 250 ng/mL of 4-methylumbelliferone-3C_4_, and 500 ng/mL of 4-methylumbelliferone glucuronide, and vortexed for 30s. After 30min of equilibration, the sample was buffered with 300 μL of 1.0 M ammonium acetate (pH 5.0; 7.7 g of ammonium acetate dissolved in 100 mL of Milli-Q water and 6 mL of acetic acid was added), and then added by 20μL of β-glucuronidase/sulfatase, followed by incubation for overnight at 37℃.

After hydrolysis, 0.5 mL of 1.0 M formic acid (3.93 mL of formic acid dissolved in 100 mL of Milli-Q deionized water) was added to stop the enzyme activity. The urine sample was loaded on an Oasis HLB solid-phase extraction (SPE) cartridge (60 mg/3mL, Waters, Milford, MA), previously prepared by conditioning with 5 mL of methanol, followed by 5 mL of Milli-Q deionized water at a flow rate of 1 mL/min. The cartridge was successively washed by 3mL of deionized water and 3mL of 40% methanol aqueous solution (v/v) at a flow rate of 0.5 mL/min, followed by being under vacuum for 10 min. The BPA was eluted with 5 mL of methanol at a flow rate of 0.5 mL/min. The methanol eluate was concentrated to dryness at a 45℃ water bath under a gentle stream of nitrogen, and reconstituted in 0.5mL of 20% acetonitrile aqueous solution (v/v). The final solution was transferred into a 2 mL amber glass vial and kept at 4℃ until analysis.

**Instrumental Analysis**

A Waters Acquity UPLC coupled with a Waters Xevo TQ triple quadrupole mass spectrometer (Beverly, MA, USA) was used for the measurement of BPA. Ten microliters of the final solution were injected onto an Acquity UPLC HSS T3 column (100mm×2.1mm×1.8µm). BPA was eluted by water (A) and acetonitrile (B) at a flow rate of 300µL/min. The gradient of organic modifier (B) varied linearly as follows: 0-0.2min, 5%B; 0.2-1.0min, 25%B; 1.0-6.0min, 55%B; 6.0-7.0min, 100%B; 7.0-8.0min, 5%B; 8.0-10.0min, 5%B. The column temperature was set to 40℃.

BPA was analyzed under negative ion (NI) mode. Nitrogen was used as the nebulization gas. The gas flow was set to 800L/h at a temperature of 450℃. The cone gas was set to 40 L/h, and the source temperature set to 120℃. The capillary voltage was set to 2.8 KV, and the sampling cone voltage was set to 45V. The multiple reaction monitoring (MRM) mode was used, and the MRM transitions monitored were 227.1>211 (quantitative ion) and 227.1>133.1 (qualitative ion) for BPA, and 241.2>222.1(quantitative ion) and 241.2>142.1(qualitative ion) for BPA-d_16_. The collision energy (CE) for both BPA and BPA-d_16_ was 28 eV.

**Quality Assurance/Quality Control**

For each batch of 20 samples analyzed, two procedural blanks and a pair of matrix spiked samples were processed. An internal standard calibration curve of four concentration levels, ranging from 0.2 ng/mL to 50 ng/mL, was prepared every batch. As a check for instrumental drift in response factors and carry-over of BPA from sample to sample, a midpoint calibration standard and a procedural blank were injected every 10 samples, respectively. The correlation coefficient of calibration curves was above 0.995. BPA was not detected in all procedural blanks. The matrix effect, expressed as the extent of suppression of BPA signal intensity, was 18.2%. The average recoveries of BPA from spiked samples varied between 86% and 108% with relative standard deviation (RSD) of 9.7%. The limit of quantitation (LOQ) and limit of detection (LOD) was 0.2 ng/mL and 0.07 ng/mL, respectively. The average recovery of 4-methylumbelliferone spiked in all samples was between 92%-109%, which indicated that BPA glucuronide was completely deconjugated by β-glucuronidase/sulfatase.

Table S1 BMI-based criteria by age and sex for screening of overweight and obese school children proposed by Working Group on Obesity in China (WGOC) in 2005

| Age(years) | Boys | | | Girls | | |
| --- | --- | --- | --- | --- | --- | --- |
|  | Normal | Overweight | Obesity | Normal | Overweight | Obesity |
| 7- | 13.5-17.3 | 17.4-19.1 | ≥19.2 | 13.4-17.1 | 17.2-18.8 | ≥18.9 |
| 8- | 13.7-18.0 | 18.1-20.2 | ≥20.3 | 13.5-18.0 | 18.1-19.8 | ≥19.9 |
| 9- | 13.9-18.8 | 18.9-21.3 | ≥21.4 | 13.6-18.9 | 19.0-20.9 | ≥21.0 |
| 10- | 14.2-19.5 | 19.6-22.4 | ≥22.5 | 13.9-19.9 | 20.0-22.0 | ≥22.1 |
| 11- | 14.5-20.2 | 20.3-23.5 | ≥23.6 | 14.3-21.0 | 21.1-23.2 | ≥23.3 |
| 12- | 14.9-20.9 | 21.0-24.6 | ≥24.7 | 14.8-21.8 | 21.9-24.4 | ≥24.5 |
| 13- | 15.5-21.8 | 21.9-25.6 | ≥25.7 | 15.7-22.5 | 22.6-25.5 | ≥25.6 |
| 14- | 16.2-22.5 | 22.6-26.3 | ≥26.4 | 16.4-22.9 | 23.0-26.2 | ≥26.3 |
| 15- | 16.8-23.0 | 23.1-26.8 | ≥26.9 | 17.0-23.3 | 23.4-26.9 | ≥26.9 |
| 16- | 17.3-23.4 | 23.5-27.3 | ≥27.4 | 17.5-23.6 | 23.7-27.3 | ≥27.4 |
| 17- | 17.7-23.7 | 23.8-27.7 | ≥27.8 | 17.7-23.7 | 23.8-27.6 | ≥27.7 |
| 18 | 18.1-23.9 | 24.0-27.9 | ≥28.0 | 17.9-23.9 | 24.0-27.9 | ≥28.0 |

Table S2 Multiple linear regression analyses for the associations between daily intake estimates (μg/day) and body mass index (BMI, Kg/m^2^) among study participants

| Group | Crude analysis ^a^ | Adjusted model 1^b^ | Adjusted model 2^c^ |
| --- | --- | --- | --- |
|  | β ^d^ (95% CI) | β (95% CI) | β (95% CI) |
| All | 0.027 (0.011,0.042) | 0.017 (0.003,0.030) | 0.015 (0.001,0.030) |
|  | P=0.001 | P=0.016 | P=0.036 |
| Age (year) |  |  |  |
| 8-11 | 0.010 (-0.010,0.030) | 0.020 (0.001,0.038) | 0.017 (-0.002,0.037) |
|  | P=0.330 | P=0.041 | P=0.081 |
| 12-15 | 0.013 (-0.012,0.039) | 0.020 (-0.003,0.044) | 0.019 (-0.005,0.044) |
|  | P=0.306 | P=0.088 | P=0.118 |
| Sex |  |  |  |
| Female | 0.027 (0.006,0.047) | 0.017 (-0.001,0.034) | 0.013 (-0.006,0.031) |
|  | P=0.011 | P=0.051 | P=0.178 |
| Male | 0.027 (0.003,0.051) | 0.016 (-0.006,0.037) | 0.018 (-0.004,0.040) |
|  | P=0.029 | P=0.150 | P=0.115 |

Abbreviation: CI (confidence interval)

^a^, Included a variable of daily intake estimate corrected for specific gravity.

^b^, Not corrected daily intake estimates by specific gravity and included the covariables of age and sex.

^c^, Corrected daily intake estimates by specific gravity and included the covariables of age and sex.

^d^, Regression coefficient.
